# Integrin α6β4 Confers Doxorubicin Resistance in Cancer Cells by Suppressing Caspase-3–Mediated Apoptosis: Involvement of N-Glycans on β4 Integrin Subunit

**DOI:** 10.3390/biom13121752

**Published:** 2023-12-06

**Authors:** Yoshinobu Kariya, Jianguo Gu, Yukiko Kariya

**Affiliations:** 1 Department of Biochemistry, Fukushima Medical University, Fukushima City 960-1295, Japan; 2 Division of Regulatory Glycobiology, Institute of Molecular Biomembrane and Glycobiology, Tohoku Medical and Pharmaceutical University, Komatsushima 981-8558, Japan; jgu@tohoku-mpu.ac.jp; 3 Medical-Industrial Translational Research Center, Fukushima Medical University, Fukushima City 960-1295, Japan

**Keywords:** α6β4 integrin, doxorubicin, chemoresistance, caspase-3, N-acetylglucosaminyltransferase III (GnT-III), bisecting GlcNAc

## Abstract

Drug resistance is a major obstacle to successful cancer treatment. Therefore, it is essential to understand the molecular mechanisms underlying drug resistance to develop successful therapeutic strategies. α6β4 integrin confers resistance to apoptosis and regulates the survival of cancer cells; however, it remains unclear whether α6β4 integrin is directly involved in chemoresistance. Here, we show that α6β4 integrin promotes doxorubicin resistance by decreasing caspase-3–mediated apoptosis. We found that the overexpression of α6β4 integrin by the β4 integrin gene rendered MDA-MB435S and Panc-1 cells more resistant to doxorubicin than control cells. The acquired resistance to doxorubicin by α6β4 integrin expression was abolished by the deletion of the cytoplasmic signal domain in β4 integrin. Similar results were found in MDA-MB435S and Panc-1 cells when N-glycan-defective β4 integrin mutants were overexpressed or bisecting GlcNAc residues were increased on β4 integrin by the co-expression of N-acetylglucosaminyltransferase III with β4 integrin. The abrogation of α6β4 integrin-mediated resistance to doxorubicin was accompanied by reduced cell viability and an increased caspase-3 activation. Taken together, our results clearly suggest that α6β4 integrin signaling plays a key role in the doxorubicin resistance of cancer cells, and N-glycans on β4 integrin are involved in the regulation of cancer cells.

## 1. Introduction

Despite recent advances in cancer treatment, drug resistance remains a major obstacle to successful cancer therapy. Many tumors are intrinsically resistant or become resistant to anticancer drugs during treatment [1,2,3]. Drug resistance can cause treatment failure in more than 90% of patients with metastatic cancer [4], resulting in poor patient prognosis and survival. Therefore, it is essential to understand the molecular mechanisms underlying drug resistance to develop successful therapeutic strategies.

Integrins are α/β heterodimeric, transmembrane, glycoprotein receptors for extracellular matrix (ECM) molecules, and regulate cellular signaling that directs many physiological and pathological processes, such as development, immunity, wound healing, fibrosis, and cancer [5,6]. Recent studies have shown that some integrins play key roles in drug resistance, especially in multiple myeloma [1,6,7]. For example, myeloma cells induce resistance to doxorubicin (DOX) by preventing apoptosis through the α4β1 and α5β1 integrins [8]. Integrin-mediated drug resistance has been observed not only in hematological malignancies but also in solid tumors [9]. The interaction of αvβ3 integrin with osteopontin activates the FAK signaling pathway to suppress apoptosis, resulting in epidermal growth factor-tyrosine kinase inhibitor (EGFR-TKI) resistance in non-small cell lung cancer with EGFR-mutation [10]. Also, multidrug-resistant breast cancer cells were sensitized to DOX and paclitaxel by the suppression of αvβ6 integrin using shRNAs targeting β6 integrin, which downregulated P-glycoprotein, a drug efflux transporter encoded by the MDR gene [11]. Therefore, integrins are considered a potential therapeutic target for overcoming resistance to chemotherapy [6].

One of the integrin receptors, α6β4 integrin, which is a laminin receptor composed of α6 and β4 integrin subunits, is highly expressed in various tumor types. α6β4 integrin expression is associated with poor prognosis of cancer patients and aggressive behavior in several cancers [12,13]. Many studies have shown that α6β4 integrin plays a crucial role in tumor progression by promoting cancer cell adhesion, migration, invasion, proliferation, tumorigenesis, as well as metastasis [13,14].

Recent studies have shown that the functional activities of α6β4 integrin are modulated by N-glycosylation on β4 integrin [15,16]. N-glycosylation is one of the major post-translational modifications of proteins, and modulates protein stability, folding, as well as functions [17,18]. The alteration in N-glycosylation is one of the hallmarks of cancer, which is mainly due to the dysregulation of glycosyltransferases, and is often associated with the malignant phenotype of cancer cells, which are drug resistant and metastatic. The deletion of all five N-glycosylation sites on β4 integrin, or the introduction of bisecting GlcNAc to N-glycans on β4 integrin by N-acetylglucosaminyltransferase III (GnT-III encoded by the MGAT3 gene) overexpression, suppresses cancer cell migration and tumorigenesis [15,16], demonstrating the pivotal role of N-glycans in α6β4 integrin-mediated tumor progression.

α6β4 integrin confers resistance to apoptosis and regulates the survival of cancer cells [19,20,21]. Thus, α6β4 integrin is predicted to influence the chemoresistance of cancer cells. In fact, α6β4 integrin contributes to gefitinib resistance in gastric cancer cells by cross-talk with EGFR [22]. Also, α6β4 integrin signaling promotes resistance to anti-ErbB2 therapy in a mouse model of ErbB2-induced mammary carcinoma [23]. However, it remains poorly understood how α6β4 integrin contributes to chemoresistance. Here, we show that α6β4 integrin promotes DOX resistance by decreasing caspase-3-mediated apoptosis, which is regulated by α6β4 integrin signaling and N-glycans on β4 integrin.

## 2. Materials and Methods

### 2.1. Plasmids and Reagents

Retroviral LZRS and lacZ-LZRS blast expression vectors were a gift from Dr. M Peter Marinkovich (Stanford University, Stanford, CA, USA). LZRS blast expression vectors encoding human wild type β4 integrin (WTβ4), a cytoplasmic domain-truncated β4 integrin mutant (∆CTβ4; residues 1–1217), an N-glycosylation site-defective β4 integrin mutant (∆Nβ4; ∆N^327^, ∆N^491^, ∆N^579^, ∆N^617^, and ∆N^695^), and GnT-III were prepared as previously described [15,24,25]. A retroviral lacZ-LZRS blast expression vector was used as a control. DOX was obtained from WAKO (Osaka Japan, #040-21521). Dimethyl sulfoxide (DMSO) was purchased from Nacalai Tesque (Kyoto, Japan, #13407-45).

### 2.2. Cell Culture

Modified human 293 phoenix cells were a gift from Dr. M Peter Marinkovich (Stanford University, Stanford, CA, USA). Human melanoma MDA-MB435S cells were obtained from the American Type Culture Collection (ATCC). These cells were maintained in Dulbecco’s modified Eagle’s medium (DMEM; WAKO, #043-30085), supplemented with 10% fetal bovine serum (FBS), penicillin, and streptomycin sulfate (WAKO, #168-23191). Panc-1 (human pancreatic cancer) cells were obtained from the RIKEN BRC through the National Bio-Resource Project of the MEXT, Japan, and were grown in RPMI-1640 (WAKO, #189-02025), supplemented with 10% FBS, penicillin, and streptomycin sulfate, 2.5 g/L D (+)-glucose (WAKO, #79-05511), 1 mM sodium pyruvate (WAKO, #190-14881), and 10 mM HEPES. Retroviruses were produced from 293 phoenix cells transfected with LZRS blast retroviral vectors using Lipofectamine LTX & Plus reagent (Invitrogen, Waltham, MA, USA, #15338-030). Cells were then selected with 5 µg/mL puromycin (Sigma-Aldrich, St. Louis, MO, USA, #P8833). Viral supernatants were passed through a 0.45 µm filter and stored at −80 °C until use. One day before viral infection, 4 × 10^5^ cells were plated in 3 mL growth medium in a six-well plate (BD Transduction Laboratories, Franklin Lakes, NJ, USA, #353846). Before infection, cells were treated with 5 µg/mL polybrene (Sigma-Aldrich, #10768-9) for 15 min, and then the growth medium was replaced with viral supernatant containing 5 µg/mL polybrene. For infection, the plate was centrifuged at 200× *g* for 1 h at 32 °C using a Hitachi CR22N centrifuge machine with an R5S4 rotor (Ibaraki, Japan), and the viral supernatant was replaced with a fresh growth medium. To establish a stable cell line, cells were selected with 10 µg/mL blasticidin S (Calbiochem, San Diego, CA, USA, #203350). Cell morphology was observed and photographed under an IX71 phase-contrast microscope (Olympus, Tokyo, Japan).

### 2.3. Immunoprecipitation and Western Blotting

For the preparation of cell lysates, cells were washed twice with cold PBS and then solubilized with lysis buffer [1% Triton X-100, 20 mM Tris-HCl (pH 7.4), 150 mM NaCl, 5 mM EDTA] containing a protease inhibitor cocktail (Nacalai Tesque, #25955-24). After incubation for 20 min on ice, the samples were centrifuged (20,400× *g*, 4 °C, 20 min) and the resulting supernatant was used as a cell lysate sample. The protein concentration of cell lysate samples was determined using a protein assay kit (Nacalai Tesque, #29449-44). For immunoprecipitation, protein G-Sepharose Fast Flow beads were added to cell lysates and rotated at 4 °C for 30 min to remove the non-specific binding proteins to the beads. After centrifugation (12,000× *g*, 4 °C, 20 s), an anti-β4 integrin antibody (Merck Millipore, Millipore, CA, USA, #MAB1964, clone 3E1) was added to the resultant supernatant and rotated at 4 °C for 2 h. Then, protein G-Sepharose was added to the antigen–antibody complex, and the samples were further rotated at 4 °C for 3 h. The Immunoprecipitated proteins were washed five times with STEN buffer [50 mM Tris-HCl (pH 7.5), 150 mM NaCl, 2 mM EDTA, 0.2% NP40 (*v*/*v*)], suspended in a reducing sample buffer (WAKO, #196-16142), and heated at 95 °C for 5 min. Then, the heated samples were centrifuged (12,000× *g*, 4 °C, 20 s) and the resulting supernatant was subjected to SDS-PAGE and Western blotting analyses.

For the Western blot analysis, proteins were separated using the SDS-PAGE procedure under reducing conditions and transferred onto nitrocellulose membranes. The membranes were blocked with 5% non-fat milk in TBS containing 0.1% (*v*/*v*) Tween 20 (TBS-T) at room temperature for 1 h, washed three times with TBS-T for 5 min, and incubated with primary antibodies at room temperature for 1 h or at 4 °C overnight. Primary antibodies against the following proteins were used: mouse monoclonal antibodies against α-tubulin (SIGMA, #T9026, clone DM1A, 1:5000) and P-glycoprotein (Calbiochem, #517310, clone C219, 1:1000), and rabbit polyclonal antibodies against cleaved caspase-3 (Cell Signaling, Danvers, MA, USA, #9661, 1:1000) and β4 integrin (Santa Cruz Biotechnology, Dallas, TX, USA, #sc-9090, 1:1000). Biotinylated PHA-E4 lectin was obtained from Seikagaku Biobusiness Corporation (Tokyo, Japan, #300425, 1:1000). For antibody detection, the membranes were washed three times with TBS-T for 5 min and incubated with horseradish peroxidase-conjugated horse anti-mouse IgG (Cell Signaling Technology, #7076, 1:5000) or goat anti-rabbit IgG antibodies (Promega, Madison, WI, USA, #W401B, 1:5000; Cell Signaling, #7074, 1:5000) antibodies for 1 h at room temperature. To detect biotinylated lectins, membranes were incubated with horseradish peroxidase-conjugated streptavidin (Thermo Fisher Scientific, Waltham, MA, USA, #21126, 1:5000) for 1 h at room temperature. After washing three times with TBS-T for 5 min, immunoreactive bands were visualized by a Trident Femto-ECL reagent (Irvine CA, USA, GeneTex, #GTX14698) or an ImmunoStar LD reagent (WAKO, #292-69903) and imaged using Imager and Image Saver 6 software (ATTO, Tokyo, Japan, #AE-9300H-CP). Densitometry was performed using ImageJ.JS software 1.53m.

### 2.4. Cell Viability Assay

Cells were plated at a density of 2 × 10^4^ cells/well (MDA-MB435S cells) or 2.5 × 10^4^ cells/well (Panc-1 cells) on a 96-well plate (Sumitomo Bakelite, Tokyo, Japan, #638-28481 or As One, Osaka, Japan, #2-8588-05) in 100 µL of growth medium and incubated at 37 °C in the presence of 5% CO_2_. After incubation for 24 h, the growth medium was replaced with DMSO solvent or DOX-containing growth medium, and the cells were further incubated at 37 °C in the presence of 5% CO_2_. After incubation for 24 h, 10 µL of Cell Counting Kit-8 (DOJINDO, Kumamoto, Japan, #343-07623) solution was added to each well of the plate, and the cells were further incubated at 37 °C for 2 h in the presence of 5% CO_2_. The color intensity was measured at 450 nm/570 nm using a microplate reader (Bio-Rad, Hercules, CA, USA, model 680).

### 2.5. FACS Analysis

For the analysis of the cell surface expression of α6β4 integrin, cells were washed twice with PBS and then incubated with 0.25% trypsin/PBS with 1 mM EDTA. After quenching trypsinization with a growth medium, the cells were washed twice with cold PBS that contained 1 mM EDTA (PBS/EDTA) and then suspended in PBS/EDTA. The cells were then incubated with primary antibodies on ice for 30 min. Primary antibodies against the following proteins were used: a mouse monoclonal antibody against β4 integrin (BioLegend, San Diego, CA, USA, #327802, clone 58XB4, 1:200), rat monoclonal antibodies against β4 integrin (BD Transduction Laboratories, #555719, clone 439-9B, 1:200) and α6 integrin (Santa Cruz Biotechnology, #sc-19622, clone GoH3, 1:200). After washing once with PBS/EDTA, the cells were incubated with an Alexa Fluor 488-conjugated goat secondary anti-mouse IgG antibody (Invitrogen, #A11029, 1:500) or an Alexa Fluor 546-conjugated goat secondary anti-rat IgG antibody (Invitrogen, #A11081, 1:500). After incubation on ice for 15 min, the cells were washed three times with PBS/EDTA. Then, the cells were analyzed with flow cytometry using FACSCalibur and CellQuestPro software version 5.2 (BD Transduction Laboratories). At least 10,000 events were analyzed for each sample.

### 2.6. Statistics and Reproducibility

Results are expressed as mean ± SEM and represent at least three independent experiments for all studies. Statistical comparisons were made between two groups using an unpaired two-tailed Student’s *t*-test and among groups using a one-way ANOVA test followed by Tukey’s post hoc test using GraphPad Prism Version 5.0a and SPSS Statistics 26 software. A *p* value of <0.05 was considered statistically significant. The Western blotting and micrograph results are representative images of three independent experiments with similar results.

## 3. Results

### 3.1. α6β4 Integrin Expression Is Involved in the Acquisition of DOX Resistance

α6β4 integrin is a cell adhesion molecule that induces anti-apoptosis and pro-survival signals [20,21]. Although such signals are often associated with drug resistance, little is known about the effect of α6β4 integrin expression on drug resistance in cancer cells.

**Figure 1:**
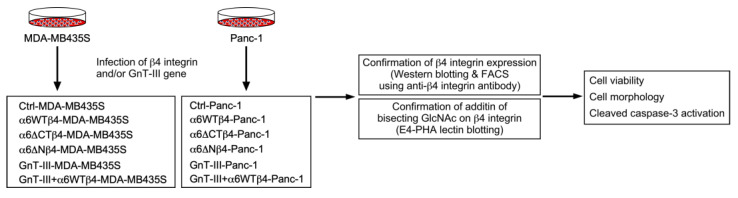
Experimental workflow. To investigate the effect of α6β4 integrin expression on drug resistance, MDA-MB435S and Panc-1 cells were retrovirally transduced with β4 integrin and/or GnT-III genes. Then, the established cells were characterized by Western blotting, FACS, and E4-PHA lectin blotting analyses. Finally, the cell viability, morphology, and caspase-3 activation were examined.

**Figure 2:**
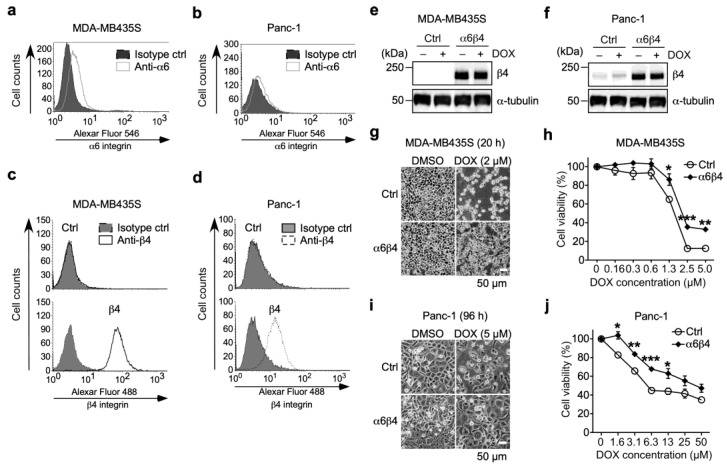
α6β4 integrin expression promotes resistance to doxorubicin. (**a**,**b**) FACS analysis of cell surface expression of α6 integrin in MDA-MB435S (**a**) and Panc-1 (**b**) cells. (**c**) FACS analysis of cell surface expression of β4 integrin in MDA-MB435S cells transduced with control (upper panel) or β4 integrin (lower panel). (**d**) FACS analysis of cell surface expression of β4 integrin in Panc-1 cells transduced with control (upper panel) or β4 integrin (lower panel). (**e**,**f**) Western blot analysis of β4 integrin expression in control and α6β4 integrin-expressing MDA-MB435S (**e**) and Panc-1 (**f**) cells treated with doxorubicin (DOX, +) or DMSO solvent (DOX, −). α-tubulin was used as a loading control. The unprocessed blot images are shown in Figure S2. (**g**,**i**) Representative images of control and α6β4 integrin-expressing MDA-MB435S (**g**) and Panc-1 (**i**) cells treated with DMSO solvent or DOX for 20 h and 96 h, respectively. (**h**,**j**) Cell viability of control and α6β4 integrin-expressing MDA-MB435S (**h**) and Panc-1 (**j**) cells treated with DOX for 24 h. The cell viability of cells treated with the DMSO solvent was calculated to be 100%. Unpaired two-tailed Student’s *t*-test, mean ± SEM of three independent experiments performed in triplicate. * *p* < 0.05. ** *p* < 0.01. *** *p* < 0.001.

To investigate the effect of α6β4 integrin expression on drug resistance, we used MDA-MB435S and Panc-1 cells because they endogenously express α6 integrin (Figure 1 and Figure 2a,b), whereas both cells have little endogenous β4 integrin expression (Figure 2c,d, upper panels). For the preparation of α6β4 integrin-overexpressing MDA-MB435S (α6WTβ4-MDA-MB435S) and Panc-1 (α6WTβ4-Panc-1) stable transfectants, the cells were retrovirally transduced with the β4 integrin gene (Figure 1). The FACS analyses showed that both α6WTβ4-MDA-MB435S and α6WTβ4-Panc-1 cells express β4 integrin on the cell surface (Figure 2c,d), indicating that these WTβ4 integrin-expressing cells express α6β4 integrin on the cell surface because β4 integrin can only associate with α6 integrin [6]. Also, noncomplexed integrin is degraded immediately or remains in the endoplasmic reticulum [26]. The expression levels of β4 integrin were not affected by the DOX treatment in both MDA-MB435S and Panc-1 cells [Figure 2e,f, DOX (−) versus DOX (+) in control (Ctrl) and α6β4 integrin-expressing cells]. When cells were treated with DOX, most control MDA-MB435S cells were detached from the culture dish, but α6β4 integrin-expressing MDA-MB435S cells were still attached to the culture dish (Figure 2g). The α6WTβ4-MDA-MB435S cells were significantly more viable than the control cells (Figure 2h). Similar results were obtained from the analysis using Ctrl and α6WTβ4-Panc-1 cells (Figure 2i,j). These results suggest that α6β4 integrin expression is associated with DOX resistance.

### 3.2. α6β4 Integrin Signaling Is Involved in the DOX Resistance Acquired by α6β4 Integrin Expression

**Figure 3:**
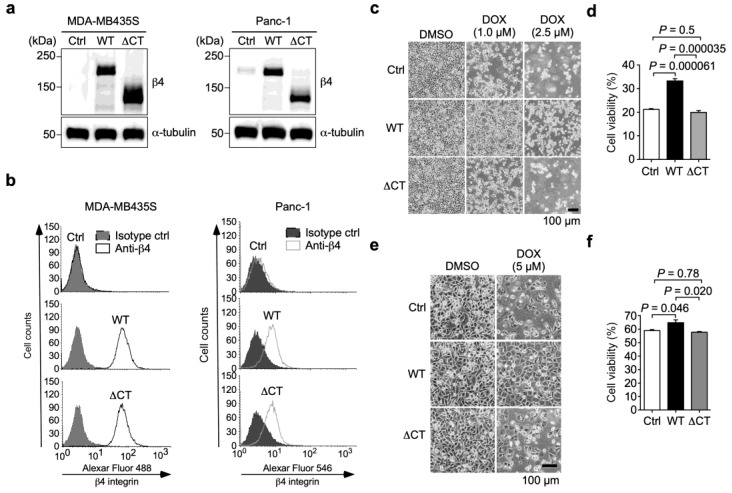
α6β4 integrin signaling is required for the doxorubicin resistance acquired by α6β4 integrin expression in MDA-MB435S and Panc-1 cells. (**a**) Western blot analyses of β4 integrin expression in MDA-MB435S and Panc-1 cells transduced with control (Ctrl), wild type (WT) β4 integrin, and β4 integrin mutant lacking cytoplasmic signaling domain (∆CT). α-tubulin was used as a loading control. The unprocessed blot images are shown in Figure S3. (**b**) FACS analysis of cell surface expression of β4 integrin in MDA-MB435S and Panc-1 cells transduced with Ctrl, WTβ4 integrin, and ∆CTβ4 integrin. (**c**) Representative images of Ctrl-, α6WTβ4-, and α6∆CTβ4-MDA-MB435S cells treated with DMSO solvent or the indicated concentration of DOX for 40 h. (**d**) Ctrl-, α6WTβ4-, and α6∆CTβ4-MDA-MDA-MB435S cells were treated with DMSO solvent or 2 µM DOX for 24 h. Cell viability of DMSO solvent-treated cells was calculated as 100%. (**e**) Representative images of Ctrl-, α6WTβ4-, and α6∆CTβ4-MDA-Panc-1 cells treated with DMSO solvent or 5 µM DOX for 96 h. (**f**) Cell viability of Ctrl-, α6WTβ4-, and α6∆CTβ4-MDA-Panc-1 cells treated with DMSO solvent or 10 µM DOX for 24 h. Cell viability of DMSO solvent-treated cells was calculated as 100%. One-way ANOVA and Tukey’s post hoc test, mean ± SEM of three independent experiments performed in triplicate.

To examine whether α6β4 integrin signaling is associated with DOX resistance, we generated a truncated β4 integrin mutant (∆CTβ4; residues 1–1217), which lacks the cytoplasmic domain required for β4 integrin signaling [14]. The mutant was then independently expressed in MDA-MB435S (α6∆CTβ4-MDA-MB435S) and Panc-1 (α6∆CTβ4-Panc-1) cells, and assessed with a Western blot analysis using an anti-β4 integrin antibody (Figure 3a). Furthermore, the FACS analyses showed that ∆CTβ4 integrin was expressed on the cell surface of both MDA-MB435S and Panc-1 cells at a similar level to WTβ4 integrin, confirming that the deletion of the cytoplasmic domain does not prevent the heterodimer formation of α6β4 integrin (Figure 3b). Following the treatment of the cells with 2.5 µM DOX for 40 h, almost all the Ctrl- and α6∆CTβ4-MDA-MB435S cells were detached from the dishes, whereas some α6WTβ4-MDA-MB435S cells remained on the dishes (Figure 3c). A cell viability assay revealed that the α6∆CTβ4-MDA-MB435S cells exhibited a reduced cell viability compared to the α6WTβ4-MDA-MB435S cells, which was comparable to the Ctrl-MDA-MB435S cells (Figure 3d). Similar results were obtained from the assays using the Ctrl-, α6WTβ4-, and α6∆CTβ4-Panc-1 cells (Figure 3e,f). These results indicate that α6β4 integrin signaling correlates with DOX resistance.

### 3.3. N-Glycans on β4 Integrin Are Involved in α6β4 Integrin-Mediated DOX Resistance

**Figure 4:**
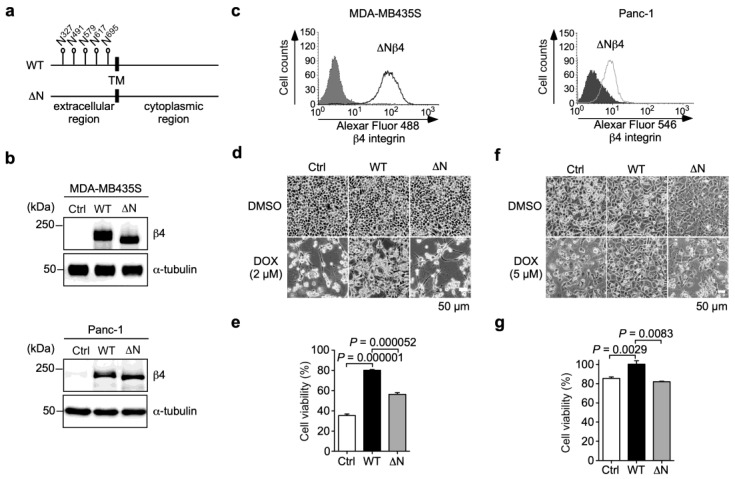
N-glycans on β4 integrin are associated with α6β4 integrin-mediated resistance to doxorubicin. (**a**) Schematic representation of WTβ4 and N-glycosylation-defective (∆N) β4 integrins. Five N-glycosylation sites on β4 integrin (Asn^327^, Asn^491^, Asn^579^, Asn^617^, and Asn^695^) are indicated by flags. TM: transmembrane region. (**b**) Western blot analyses of β4 integrin expression in MDA-MB435S and Panc-1 cells transduced with Ctrl-, WTβ4 integrin, and ∆Nβ4 integrin genes. α-tubulin was used as a loading control. The unprocessed blot images are shown in Figure S4. (**c**) FACS analysis of cell surface expression of ∆Nβ4 integrin in MDA-MB435S and Panc-1 cells stably expressing ∆Nβ4 integrin. Dark color and light color indicate isotype control and anti-β4 integrin antibody, respectively. (**d**) Representative images of Ctrl-, α6WTβ4-, and α6∆Nβ4-MDA-MB435S cells treated with DMSO solvent or 2 µM DOX for 40 h. (**e**) Cell viability of Ctrl-, α6WTβ4-, and α6∆Nβ4-MDA-MDA-MB435S cells treated with DMSO solvent or 2 µM DOX for 24 h. The cell viability of cells treated with the DMSO solvent was calculated to be 100%. One-way ANOVA and Tukey’s post hoc test, mean ± SEM of three independent experiments performed in triplicate. (**f**) Representative images of Ctrl-, α6WTβ4-, and α6∆Nβ4-MDA-Panc-1 cells treated with DMSO solvent or 5 µM DOX for 96 h. (**g**) Cell viability of Ctrl-, α6WTβ4-, and α6∆Nβ4-MDA-Panc-1 cells treated with DMSO solvent or 3.1 µM DOX for 24 h. The cell viability of cells treated with the DMSO solvent was calculated to be 100%. One-way ANOVA and Tukey’s post hoc test, mean ± SEM of three independent experiments performed in triplicate.

Our previous studies have shown that N-glycans on β4 integrin play important roles in cancer progression, including migration, invasion, proliferation, and tumorigenicity [15,16]. To evaluate whether the N-glycosylation of β4 integrin affects α6β4 integrin-induced DOX resistance, we established both MDA-MB435S and Panc-1 cells stably expressing an N-glycosylation site-defective β4 integrin mutant, ∆Nβ4 integrin, which had no N-glycan (Figure 4a). The expression of ∆Nβ4 integrin in MDA-MB435S and Panc-1 cells was confirmed using Western blot and FACS analyses. The Western blot analysis showed that the molecular size of ∆Nβ4 integrin was smaller than that of WTβ4 integrin, which was probably due to a lack of N-glycosylation (Figure 4b). The FACS analysis suggests that the lack of N-glycans on β4 integrin did not affect the cell surface expression of β4 integrin, which was associated with the heterodimer formation of β4 integrin with α6 integrin (Figure 4c). This result was consistent with our previous results obtained from human keratinocytes [25]. In the presence of DOX, the defect of N-glycans on β4 integrin markedly reduced α6β4 integrin-induced cell viability in both MDA-MB435S (Figure 4d,e; WTβ4 versus ∆Nβ4) and Panc-1 cells (Figure 4f,g; WTβ4 versus ∆Nβ4), suggesting that N-glycans on β4 integrin play a crucial role in α6β4 integrin-mediated DOX resistance.

### 3.4. α6β4 Integrin-Mediated DOX Resistance Is Abolished by the Addition of Bisecting GlcNAc to β4 Integrin

**Figure 5:**
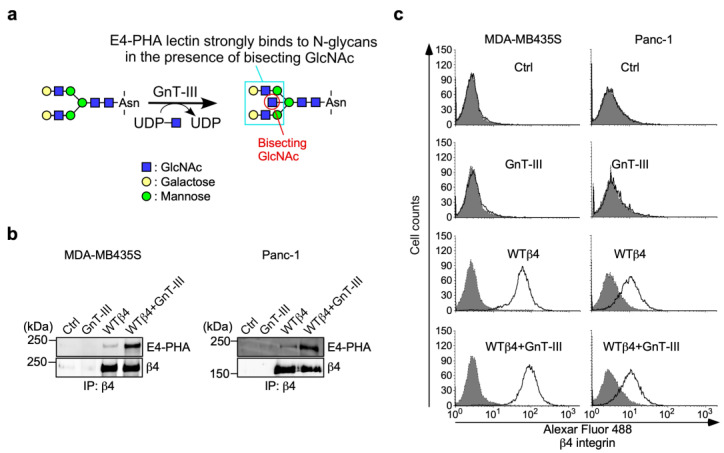
Characterization of β4 integrin in GnT-III and/or WTβ4 integrin overexpressing MDA-MB435S and Panc-1 cells. (**a**) Schematic representation of glycosylation reaction catalyzed by N-acetylglucosaminyltransferase III (GnT-III). GnT-III catalyzes the addition of bisecting GlcNAc to N-glycan. E4-PHA lectin recognizes the complex type of N-glycans containing bisecting GlcNAc. (**b**) Analysis of bisected N-glycans on β4 integrin in MDA-MB435S and Panc-1 cells with GnT-III and/or WTβ4 integrin (WTβ4). Cell lysates were immunoprecipitated with an anti-β4 integrin antibody, and then the immunoprecipitates were immunoblotted with E4-PHA lectin or an anti-β4 integrin antibody. The unprocessed blot images are shown in Figure S5. (**c**) FACS analysis of cell surface expression of β4 integrin in MDA-MB435S and Panc-1 cells with GnT-III and/or WTβ4 integrin. Dark color and light color indicate isotype control and anti-β4 integrin antibody, respectively.

To further investigate the role of N-glycans on β4 integrin in α6β4 integrin-mediated DOX resistance, we examined the effect of N-acetylglucosaminyltransferase III (GnT-III) overexpression in α6WTβ4-MDA-MB435S and α6WTβ4-Panc-1 cells on DOX resistance. GnT-III is a glycosyltransferase encoded by the MGAT3 gene that catalyzes the addition of bisecting GlcNAc to N-glycans on proteins (Figure 5a). Previous studies have shown that the expression of GnT-III is correlated with the chemoresistance of cancer cells [27,28]. Thus, we hypothesized that N-glycans containing bisecting GlcNAc (bisected N-glycans) on β4 integrin are related to α6β4 integrin-mediated DOX resistance.

To test this hypothesis, we introduced bisecting GlcNAc to N-glycans on β4 integrin by GnT-III overexpression in α6WTβ4-MDA-MB435S cells. A lectin blot analysis using E4-PHA lectin, which recognizes a complex type of N-glycans containing bisecting GlcNAc (Figure 1 and Figure 5a), demonstrated that the bisected N-glycans on β4 integrin in GnT-III-overexpressing α6WTβ4-MDA-MB435S cells were increased compared to those in α6WTβ4-MDA-MB435S cells (Figure 5b). The FACS analyses showed that GnT-III overexpression in α6WTβ4-MDA-MB435S cells did not affect the cell surface expression of β4 integrin (Figure 5c). Similar results were obtained from Panc-1 cells stably expressing GnT-III and/or WTβ4 integrin (Figure 5b,c).

**Figure 6:**
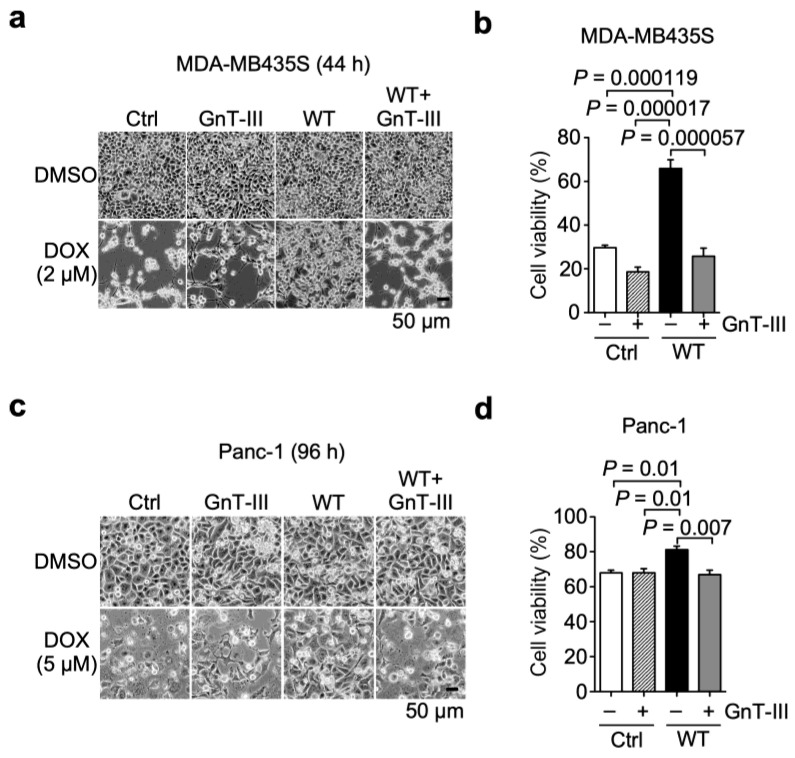
The addition of bisecting GlcNAc to N-glycans on β4 integrin decreased α6β4 integrin-mediated resistance to doxorubicin. (**a**) Representative images of Ctrl- and α6WTβ4 (WT)-MDA-MB435S cells stably expressing GnT-III treated with DMSO solvent or 2 µM DOX for 44 h. (**b**) Cell viability of the indicated MDA-MB435S cells treated with 2 µM DOX for 24 h. The cell viability of cells treated with the DMSO solvent was calculated to be 100%. One-way ANOVA and Tukey’s post hoc test, mean ± SEM of three independent experiments performed in triplicate. (**c**) Representative images of Ctrl- and α6WTβ4 (WT)-Panc-1 cells stably expressing GnT-III treated with DMSO solvent or 5 µM DOX for 96 h. (**d**) Cell viability of the indicated Panc-1 cells treated with 5 µM DOX for 24 h. The cell viability of cells treated with the DMSO solvent was calculated to be 100%. One-way ANOVA and Tukey’s post hoc test, mean ± SEM of three independent experiments performed in triplicate.

Next, we examined the effect of the addition of bisecting GlcNAc to N-glycans on β4 integrin on α6β4 integrin-mediated resistance to DOX using the GnT-III-overexpressing cells. In the presence of DOX, GnT-III overexpression decreased the number of adherent cells to dishes in α6WTβ4-MDA-MB435S cells. In contrast, almost all Ctrl- and GnT-III-overexpressing MDA-MB435S cells were detached from the dishes (Figure 6a). Consistently, the cell viability assay revealed that the overexpression of GnT-III abolished the ability of α6β4 integrin to induce DOX resistance in α6WTβ4-MDA-MB435S cells, which was comparable to Ctrl-, and GnT-III-overexpressing MDA-MB435S cells (Figure 6b). Similar results were obtained from Panc-1 cells expressing GnT-III and/or WTβ4 integrin (Figure 6c,d). Collectively, these results suggest that the bisected N-glycans on β4 integrin negatively regulate α6β4 integrin-mediated resistance to DOX and provide further evidence that N-glycans on β4 integrin play a pivotal role in the DOX resistance.

**Figure 7:**
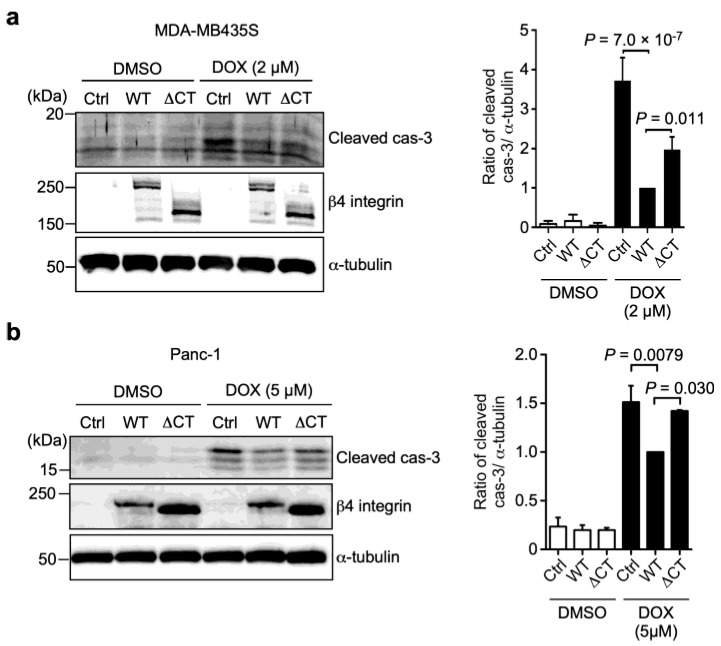
α6β4 integrin signaling suppresses DOX-induced caspase-3 activation. Western blot analysis of cleaved caspase-3 and β4 integrin in Ctrl-, α6WTβ4 (WT)-, and α6∆CTβ4 (∆CT)-MDA-MB-435S (**a**) and Panc-1 (**b**) cells treated with DMSO or DOX for 24 h. α-tubulin was used as a loading control. The ratio of cleaved caspase-3 to α-tubulin in α6WTβ4 integrin-expressing cells treated with DOX was calculated as 1.0. One-way ANOVA and Tukey’s post hoc test, mean ± SEM of three independent experiments. The unprocessed blot images are shown in Figures S6 and S7.

The prevention of apoptosis often confers drug resistance [8,29]. Therefore, we hypothesized that β4 integrin-mediated DOX resistance might occur through the prevention of apoptosis and then examined the cleavage of caspase-3, a marker of cells undergoing apoptosis (Figure 1). The treatment with DOX markedly induced the cleavage of caspase-3 in both Ctrl-MDA-MB435S and Panc-1 cells compared to the DMSO solvent treatment (Figure 7). Compared with the Ctrl cells, the expression of α6WTβ4 integrin in MDA-MB435S and Panc-1 cells markedly reduced the DOX-induced cleavage of caspase-3. In contrast, the cells expressing ∆CTβ4 integrin clearly showed an increased cleavage of caspase-3 compared to the WTβ4 integrin-expressing cells. These findings suggest that α6β4 integrin signaling induces DOX resistance by suppressing caspase-3-mediated apoptosis.

**Figure 8:**
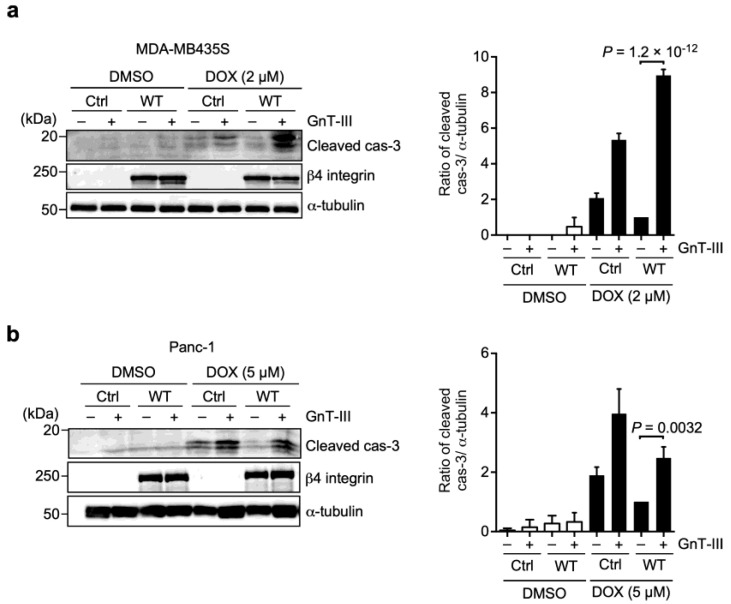
GnT-III abrogates α6β4 integrin−mediated suppression of DOX−induced caspase-3 activation. (**a**) Western blot analysis of cleaved caspase-3 and β4 integrin in Ctrl-, and α6WTβ4-MDA-MB-435S cells stably expressing GnT-III treated with DMSO solvent or 2 µM DOX for 24 h. (**b**) Western blot analysis of cleaved caspase-3 and β4 integrin in Ctrl-, and α6WTβ4-Panc-1 cells stably expressing GnT-III treated with DMSO solvent or 5 µM DOX for 48 h. α-tubulin was used as a loading control. The cleaved caspase-3 to α-tubulin ratio in α6WTβ4 integrin-expressing cells treated with DOX was calculated as 1.0. One-way ANOVA and Tukey’s post hoc test, mean ± SEM of three independent experiments. The unprocessed blot images are shown in Figures S8 and S9.

Furthermore, we tested whether the negative regulation of α6β4 integrin-mediated resistance to DOX by bisected N-glycans on β4 integrin was associated with caspase-3 activation. Indeed, the overexpression of GnT-III in α6WTβ4-MDA-MB-435S and α6WTβ4-Panc-1 cells markedly increased the cleaved caspase-3 (Figure 8). These findings demonstrate that the bisected N-glycans on β4 integrin negatively regulates α6β4 integrin-mediated resistance to DOX via caspase-3 activation and the critical role of N-glycans on β4 integrin in the process of DOX resistance.

## 4. Discussion

Drug resistance is a significant obstacle to successful cancer treatment. Elevated expression levels of specific integrins are known to be associated with drug resistance. α6β4 integrin is a component of the hemidesmosomes, while it is overexpressed in various types of cancers and plays a crucial role in tumorigenesis and metastasis. However, little is known about α6β4 integrin-mediated drug resistance. Using MDA-MB435S and Panc-1 cells expressing the β4 integrin mutant with a defective signaling domain, and the cells co-expressing β4 integrin and GnT-III, we demonstrated that α6β4 integrin confers DOX-resistance by suppressing caspase-3-mediated apoptosis through the N-glycan.

Recent studies have shown that PI3K/Akt and ERK pathways provide downstream anti-apoptotic signals and are associated with DOX resistance [30,31]. Many groups have demonstrated that α6β4 integrin activates PI3K/Akt and ERK pathways in cooperation with receptor tyrosine kinases (RTKs) [32,33]. In fact, α6β4 integrin contributes to drug resistance by regulating some RTKs, such as EGFR and ErbB2 [22,23]. Furthermore, our previous studies have shown that the overexpression of GnT-III or lack of N-glycans on β4 integrin downregulated PI3K/Akt and ERK signaling pathways [15,34]. The overexpression of β1,6GlcNAc-branched N-glycans is often observed in various tumor tissues, and the expression level is associated with malignancy and a poor prognosis [35]. Our previous studies have shown that β1,6GlcNAc-branched N-glycans are markedly increased on β4 integrin in cutaneous squamous cell carcinoma tissues compared with normal skin tissue [15,16]. The binding of galectin-3 to β1,6GlcNAc-branched N-glycans on β4 integrin induces the formation of a supramolecular complex consisting of α6β4 integrin, EGFR, and laminin-332, which in turn promotes cancer cell adhesion and migration via the activation of PI3K and ERK signaling pathways [15,34]. Therefore, it is possible that PI3K/Akt and ERK signaling pathways are involved in α6β4 integrin-induced DOX resistance via N-glycans.

N-glycans on β4 integrin are important for the localization of α6β4 integrin to lipid rafts in the plasma membrane and the interaction of α6β4 integrin with EGFR [25]. The localization of α6β4 integrin to lipid rafts activate PI3K by facilitating close interactions with EGFR [36]. Since the ∆CTβ4 integrin subunit contains N-glycosylation sites in the extracellular domain, α6∆CTβ4 integrin may associate with EGFR via N-glycans in lipid rafts and promote cell survival. Thus, α6∆CTβ4 integrin might decrease the cleaved caspase-3 level in α6∆CTβ4-MDA-MB435S cells compared to control cells (Figure 7a). However, since cell viabilities were comparable between α6∆CTβ4-MDA-MB435S and control cells (Figure 3d), it might be possible that the cleaved caspase-3 level in α6∆CTβ4-MDA-MB435S cells is enough for inducing cell death to a similar level observed in control cells.

The introduction of bisecting GlcNAc to N-glycan decreases β1,6GlcNAc-branched N-glycans [14]. Many studies have reported that decreased bisected N-glycans and increased β1,6GlcNAc-branched N-glycans were observed in various drug-resistant cells [37,38]. These results are consistent with our present results; the addition of bisecting GlcNAc to N-glycans on β4 integrin by GnT-III overexpression decreased β4 integrin-induced DOX resistance. In contrast, β1,6GlcNAc-branched N-glycans on α5β1 integrin were found to be dramatically reduced in cisplatin-resistant head and neck cancer HSC-2 cells [39]. The discrepancy could be due to several factors, including modifying different integrins and using drugs and cell lines.

Recently, we have reported that K562/ADR cells, a DOX-resistant derivative of human myeloid leukemia K562 cells, had decreased N-glycan bisection and an increased expression of a drug efflux transporter, P-glycoprotein, compared to parental K562 cells. In addition, the overexpression of GnT-III in K562/ADR cells decreased DOX resistance [27]. These results are consistent with the present finding that the overexpression of GnT-III decreased DOX resistance in α6WTβ4-MDA-MB435S and α6WTβ4-Panc-1 cells. In contrast, decreased DOX resistance in K562/ADR cells by GnT-III overexpression was caused by the downregulation of P-glycoprotein expression. However, MDA-MB435S cells did not change P-glycoprotein expression regardless of treatment with DOX and/or β4 integrin expression (Figure S1). The upregulation of P-glycoprotein is associated with multidrug resistance, and its expression is regulated by some integrins in drug-resistant cells [11]. This differential expression of P-glycoprotein might be caused by the different exposure times to DOX (long-term exposure, K562/ADR cells; short-term exposure, MDA-MB435S cells), because only the selection for drug resistance by long-term exposure overcomes the translational block of P-glycoprotein mRNA, allowing for the translation of P-glycoprotein [37]. Therefore, the underlying mechanisms of DOX resistance observed in the α6WTβ4-MDA-MB435S and α6WTβ4-Panc-1 cells used in the present study may differ from those observed in K562/ADR cells and other cells that have acquired drug resistance by long-term exposure. Thus, the present study provides new insights into the molecular mechanisms involved in the N-glycosylation of integrin-mediated drug resistance in cancer cells. A comprehensive investigation is required to understand the role of N-glycosylation modification on β4 integrin in drug resistance.

In the tumor microenvironment, the cell adhesion of tumor cells to the surrounding extracellular matrix (ECM), such as fibronectin, collagen, osteopontin, and laminin, promotes cell survival and proliferation and prevents apoptosis [40,41,42]. These effects of cell adhesion are now recognized as a major cause of intrinsic and acquired/adaptive therapy resistance, termed cell adhesion-mediated drug resistance (CAM-DR) [29]. In fact, α6β4 integrin expression promoted DOX resistance in MDA-MB435S and Panc-1 cells, although we do not know the binding ECM proteins for α6β4 integrin in the two cell lines. Furthermore, Panc-1 cells expressing low levels of β4 integrin were less sensitive to DOX treatment than MDA-MB435S cells that do not express β4 integrin. Since CAM-DR is largely mediated by non-transcriptional mechanisms, including cell proliferation, anti-apoptosis, and survival, the underlying mechanisms may be less complex compared to the resistance acquired by long-term exposure to drugs [1,29]. Therefore, the treatment strategies against α6β4 integrin-induced CAM-DR would be more efficient.

## 5. Conclusions

In conclusion, our results demonstrate that α6β4 integrin signaling plays a critical role in the doxorubicin resistance of cancer cells, and N-glycans on β4 integrin are important for the regulation of cancer cells. Although DOX is widely used in the treatment of many cancers, its clinical use is limited by cumulative, dose-dependent adverse effects, particularly cardiotoxicity [38]. In α6β4 integrin-positive tumors, the suppression of the expression of α6β4 integrin might enhance the efficacy of DOX, thereby allowing for a reduction in the DOX dosage, and thus preventing the occurrence of adverse effects. Further studies on the signaling pathways associated with α6β4 integrin-mediated DOX resistance and its role in vivo are warranted to explore the potential applications of β4 integrin and its N-glycans as therapeutic agents and as companion biomarkers in cancer therapy.

## Data Availability

All data supporting the findings in this study are included in this published article. Any additional information may be available from the corresponding author upon request.

## Supplementary Materials

The following supporting information can be downloaded at: https://www.mdpi.com/article/10.3390/biom13121752/s1, Figure S1: Western blot analysis of P-glycoprotein expression; Figure S2: Unprocessed blot images in Figure 2e,f; Figure S3: Unprocessed blot images in Figure 3a; Figure S4: Unprocessed blot images in Figure 4b; Figure S5: Unprocessed blot images in Figure 5b; Figure S6: Unprocessed blot images in Figure 7a; Figure S7: Unprocessed blot images in Figure 7b; Figure S8: Unprocessed blot images in Figure 8a; Figure S9: Unprocessed blot images in Figure 8b; Figure S10: Unprocessed blot images in Figure S1; Source data S1: The values on the graph.
