# Supplementary material for: Integrin α6β4 Confers Doxorubicin Resistance in Cancer Cells by Suppressing Caspase-3–Mediated Apoptosis: Involvement of N-Glycans on β4 Integrin Subunit
Source: Biomolecules. 2023 Dec 6;13(12):1752. doi: 10.3390/biom13121752 (PMC10741852; doi:10.3390/biom13121752)
Supplement: Supplementary file 1 [file biomolecules-13-01752-s001.zip › Figure S.pdf]

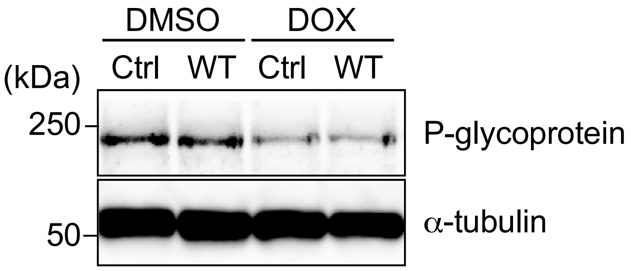

Figure S1. Western blot analysis of P-glycoprotein expression. Control (Ctrl), and WTβ4-MDA-MB435S cells were treated with solvent DMSO or 1 μM DOX for 24h, and the cell lysates were analyzed by Western blot using anti-P-glycoprotein antibody. α-tubulin was used as a loading control. Unprocessed images are shown in Figure S10.

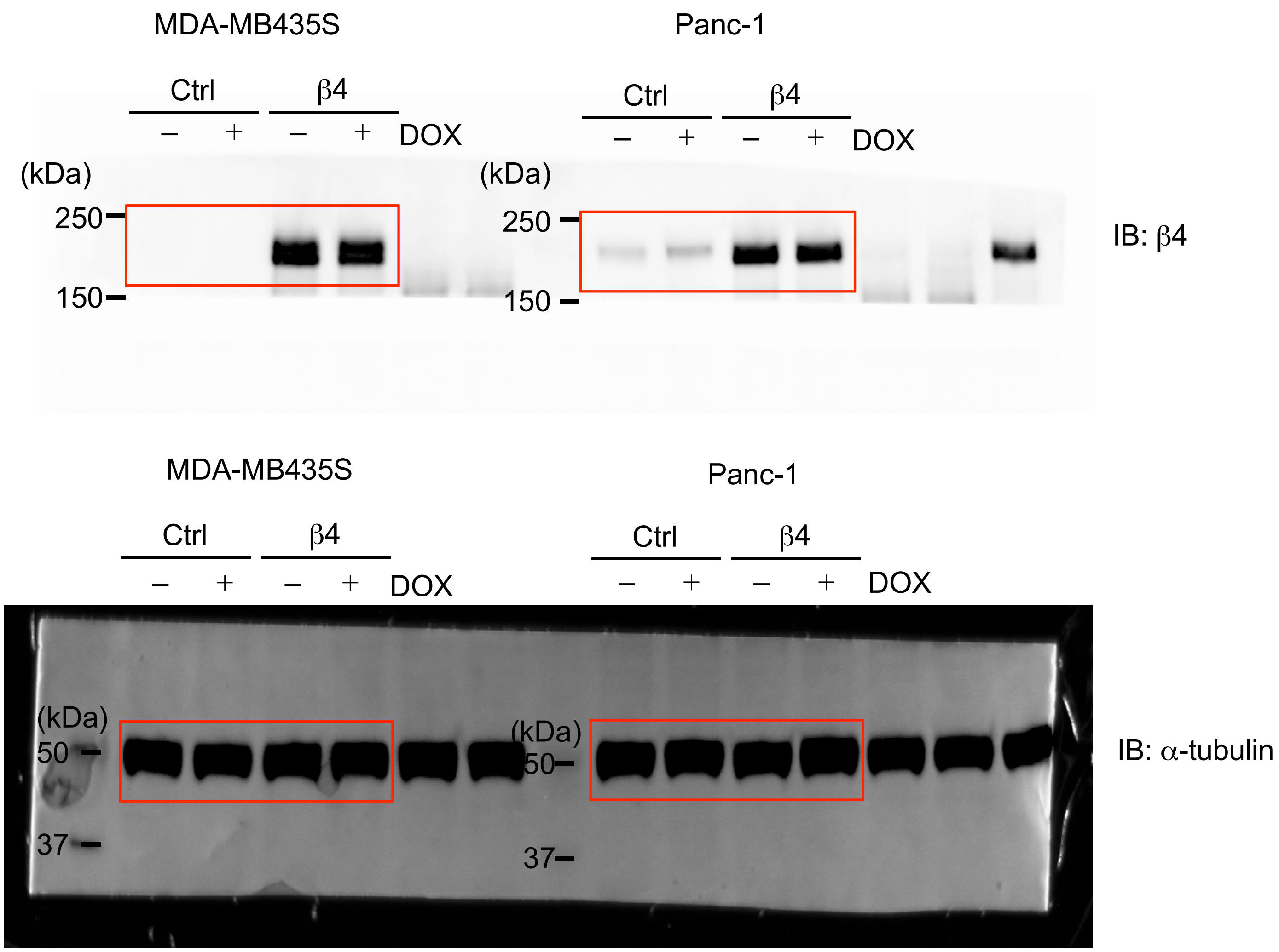

Figure S2. Unprocessed blot images in Fig. 2e and 2f.
Red square indicates cropped section.

MDA-MB435S

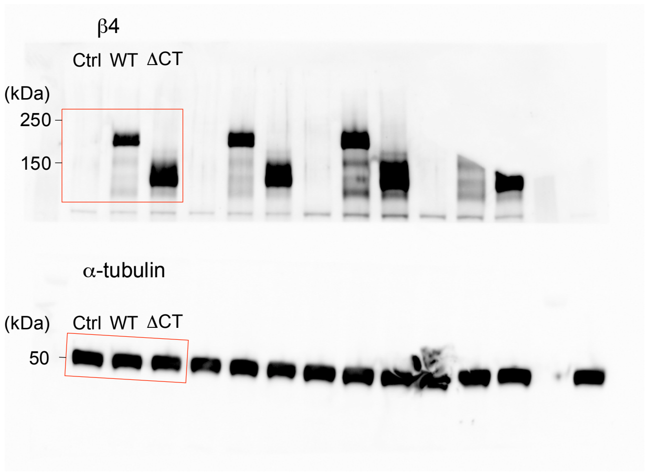

Panc-1

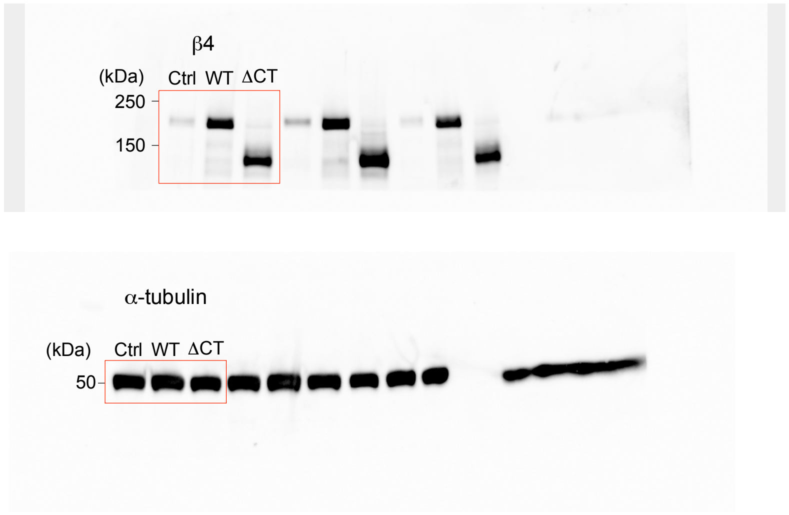

Figure S3. Unprocessed blot images in Fig. 3a.
Red square indicates cropped section.

MDA-MB435S

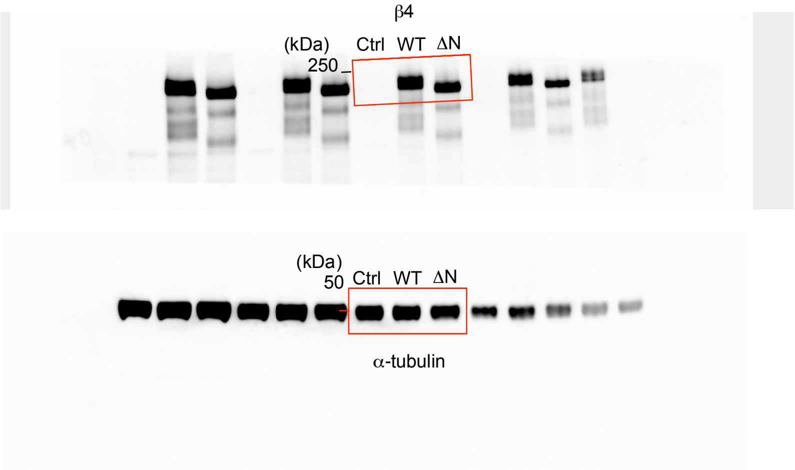

Panc-1

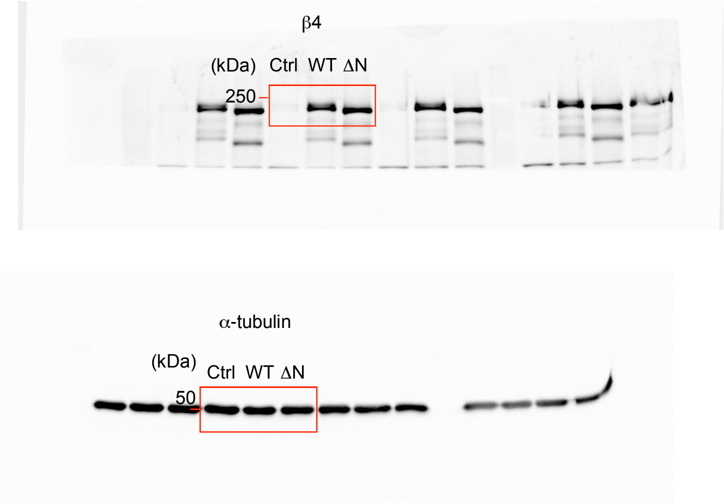

Figure S4. Unprocessed blot images in Fig. 4b
Red square indicates cropped section.

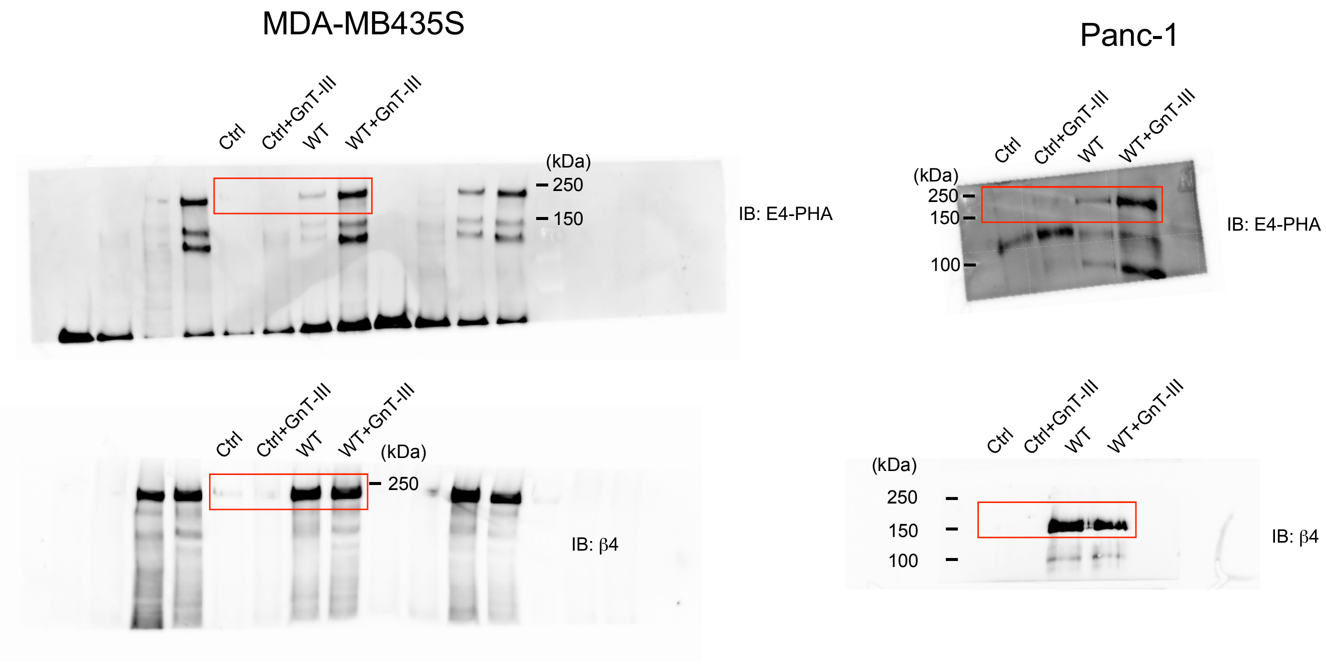

Figure S5. Unprocessed blot images in Fig 5b.
Red square indicates cropped section.

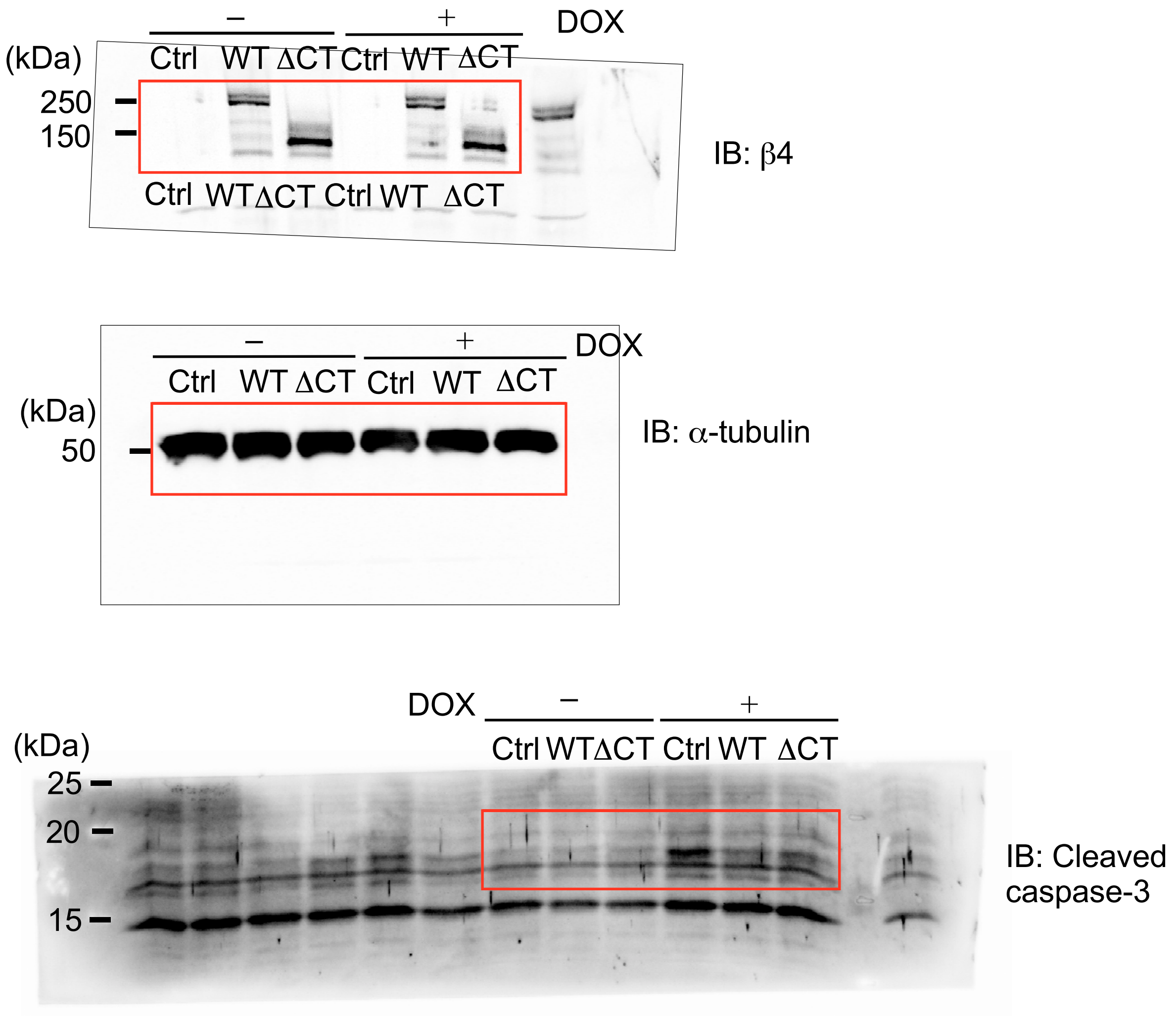

Figure S6. Unprocessed blot images in Fig. 7a.
Red square indicates cropped section.

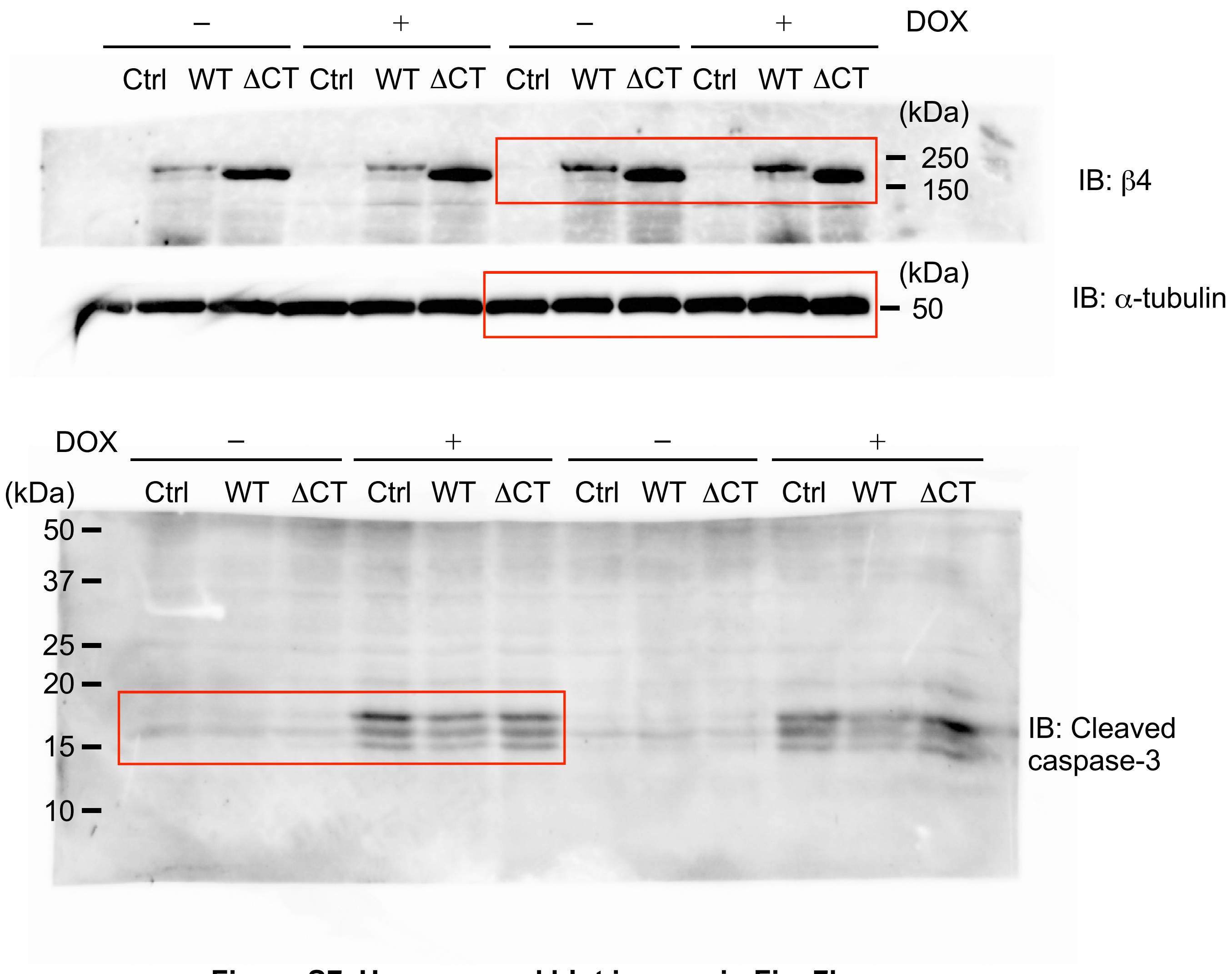

Figure S7. Unprocessed blot images in Fig. 7b.
Red square indicates cropped section.

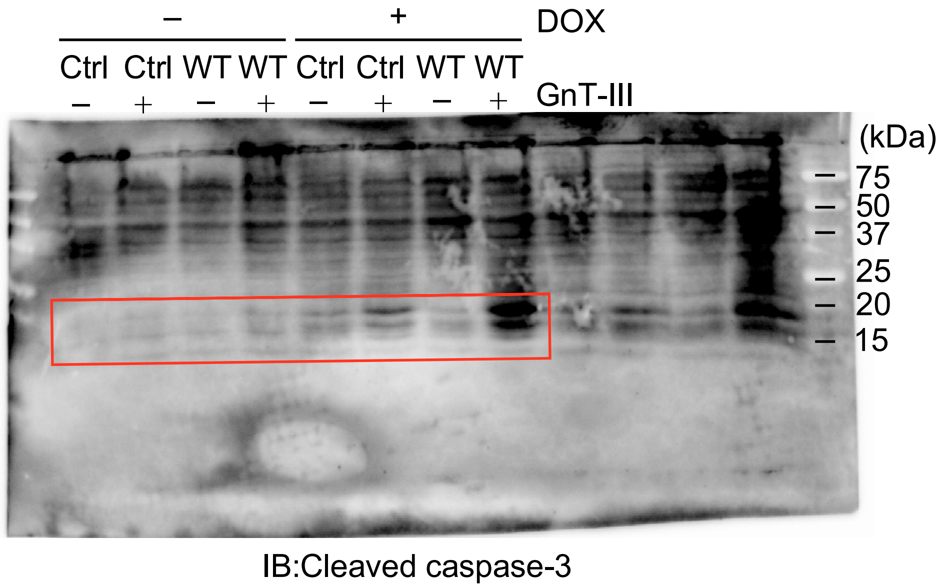

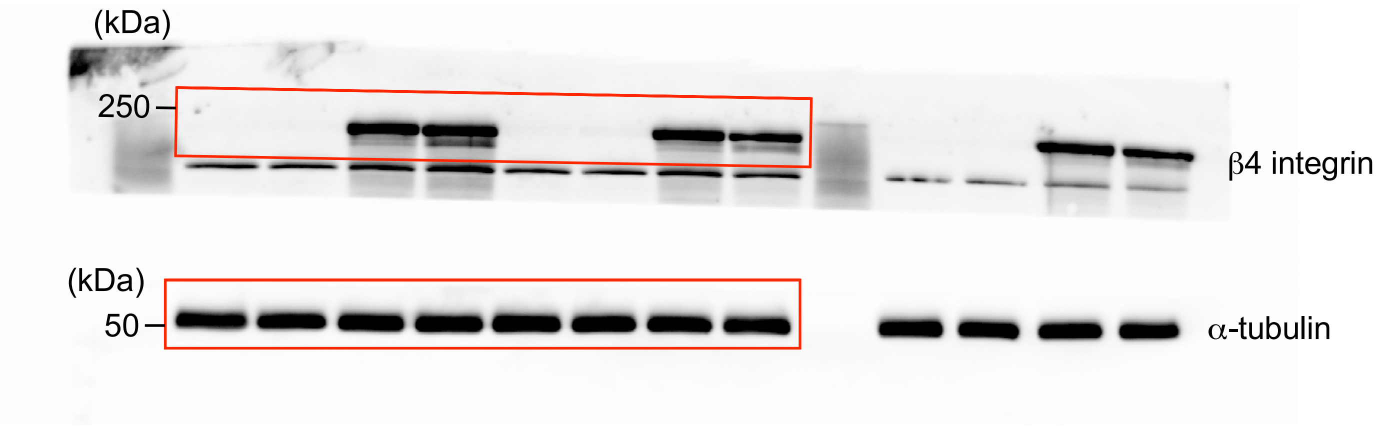

Figure S8. Unprocessed blot images in Figs. 8a.
Red square indicates cropped section.

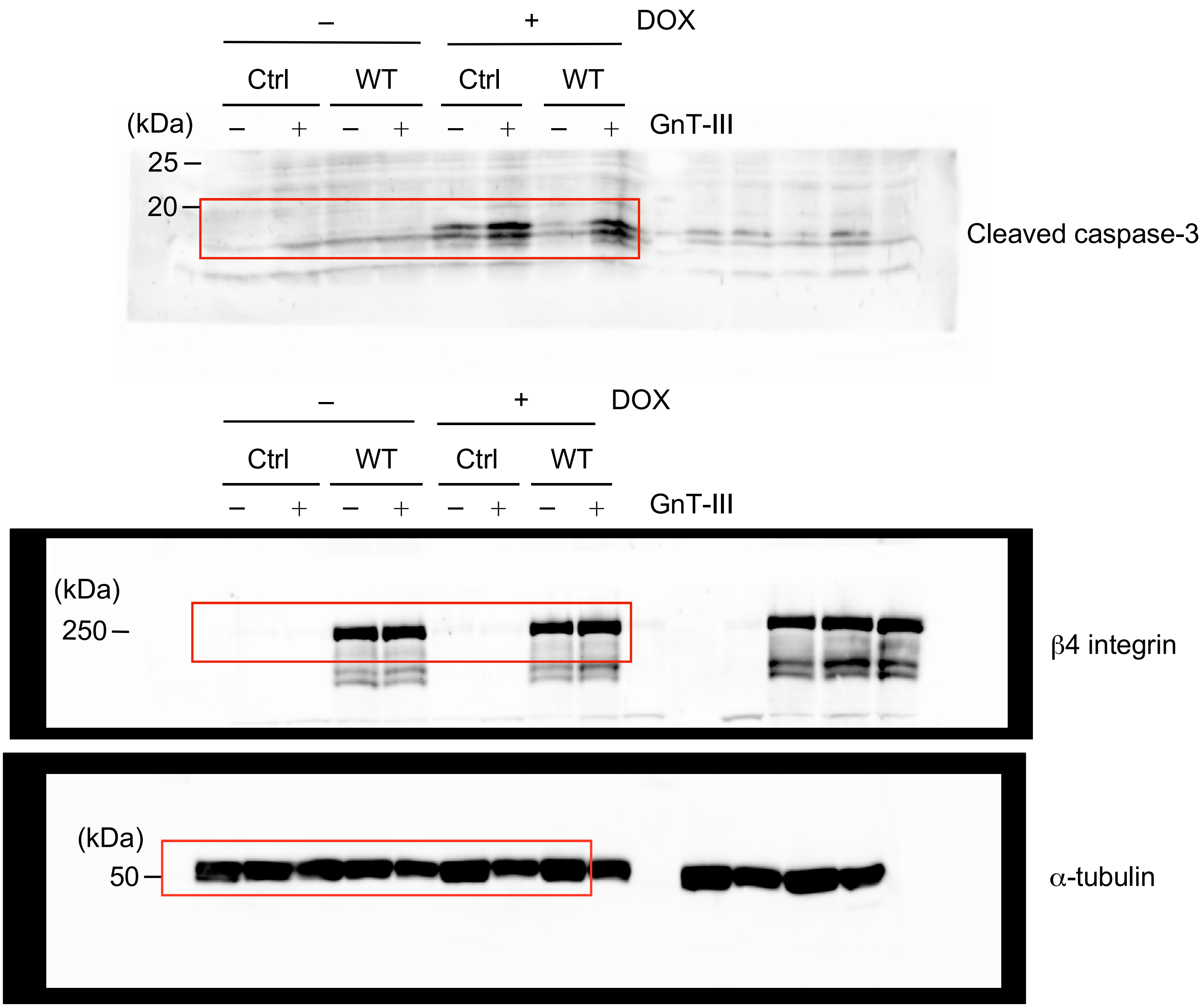

Figure S9. Unprocessed blot images in Figs. 8b.
Red square indicates cropped section.

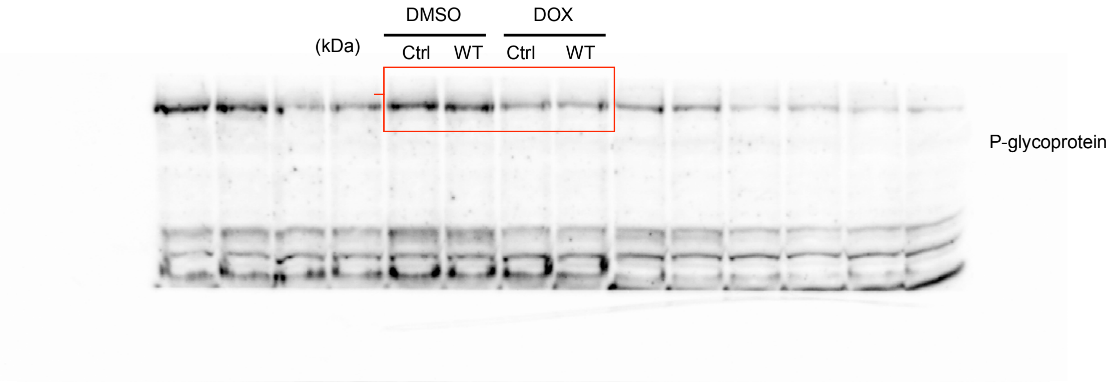

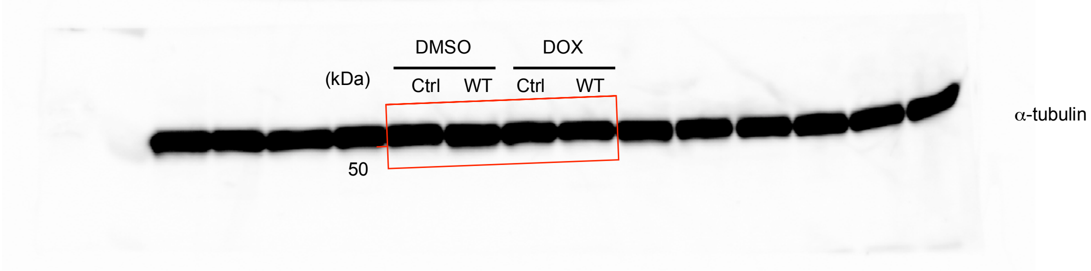

Figure S10. Unprocessed blot images in Figure S1.
Red square indicates cropped section.
